# Nucleation and annihilation of skyrmions in Mn_2_CoAl observed through the topological Hall effect

**DOI:** 10.1038/s41598-017-13211-8

**Published:** 2017-10-19

**Authors:** B. M. Ludbrook, G. Dubuis, A.-H. Puichaud, B. J. Ruck, S. Granville

**Affiliations:** 1 The MacDiarmid Institute for Advanced Materials and Nanotechnology, School of Chemical and Physical Sciences, Victoria University of Wellington, P.O. Box 600, Wellington, 6140 New Zealand; 2 The MacDiarmid Institute for Advanced Materials and Nanotechnology, Robinson Research Institute, Victoria University of Wellington, P.O. Box 33436, Lower Hutt, 5046 New Zealand

## Abstract

Magnetic skyrmions are topologically protected spin textures with great technological potential. These topologically non-trivial non-coplanar spin textures give rise to a topological Hall effect, enabling the purely electronic detection of magnetic skyrmions. We report a clear topological Hall effect in thin films of the the Heusler alloy Mn_2_CoAl, a ferromagnetic spin-gapless semiconductor, capped by a thin layer of Pd. We exploit the strong thickness- and temperature-dependence of the anomalous Hall effect in this system, tuning it to zero to enable the unambiguous measurement of the topological Hall effect, which is observed for temperatures between 3 K and 280 K. The topological Hall effect is evidence of skyrmions, and we demonstrate the simultaneous coexistence of opposite polarity skyrmions using a novel method involving minor field loops of the Hall effect.

## Introduction

The recent explosion of interest in magnetic skyrmions, particularly in thin film systems^1^, has driven interest in the electronic detection of these topological magnetic solitons. The time-varying magnetic field experienced by an electron passing through a topologically non-trivial spin texture such as a skyrmion generates an emergent magnetic field that in turn exerts an additional Lorentz force on the electron^2^. This appears as an additional transverse voltage in the Hall effect, and is known as the topological Hall effect^3,4^. This term (${\rho }_{xy}^{T}$) adds to the ordinary (${\rho }_{xy}^{O}$) and anomalous (${\rho }_{xy}^{A}$) Hall terms, so that the total Hall effect in a magnetic material with skyrmions is written

$${\rho }_{xy}={\rho }_{xy}^{O}+{\rho }_{xy}^{A}+{\rho }_{xy}^{T}.$$

The topological Hall effect has been observed in bulk and thin films of non-centrosymmetric chiral magnets such as MnSi and MnGe^5–9^. In these materials, the lack of inversion symmetry introduces a Dzyaloshinskii-Moriya interaction (DMI) that competes with ferromagnetic exchange, leading to a magnetic skyrmion phase at low temperature. The broken symmetry at the interface of a ferromagnetic thin film coupled to a heavy metal layer can also generate a DMI, and skyrmions have been observed in a number of such systems over a much wider temperature range^10–13^. The topological Hall effect is becoming an increasingly important technique for the detection of skyrmions in these systems^11,12^.

Heusler alloys represent a class of immensely tunable materials^14^. Many are ferromagnetic with high Curie temperatures and highly spin polarized band structures, making them attractive for spintronic device applications^15^. Skyrmions may be moved by spin-transfer-torque using current densities 5–6 orders of magnitude lower than conventional domain walls^16^. The ability to generate skyrmions in these materials will expand device possibilities substantially, particularly with regards to racetrack memory & its variants^17,18^. A topological hall effect has been recently observed in thin films of Heusler Mn_2_RhSn below 100 K^19^. However, most Heuslers have inversion symmetry, so the path to skyrmions will require establishing an interfacial DMI in multilayer films. The DMI must be strong enough that it is non-negligible relative to the bulk ferromagnetic exchange interaction that seeks to align the spins co-linearly. Here, we study the Heusler alloy Mn_2_CoAl, a spin-gapless semiconductor^20,21^. We show that thin trilayer films of sputtered Mn_2_CoAl, sandwiched between a 2 nm thick MgO layer and a 2.5 nm thick Pd capping layer, show a clear topological Hall effect from below 3 K to near ambient temperature, from which we infer the presence of skyrmions. We employ minor field loops in the Hall effect measurements to explore the skyrmion nucleation and annihilation dynamics, and demonstrate the coexistence of opposite polarity skyrmions.

## Topological Hall Effect in Mn_2_CoAl

**Figure 1:**
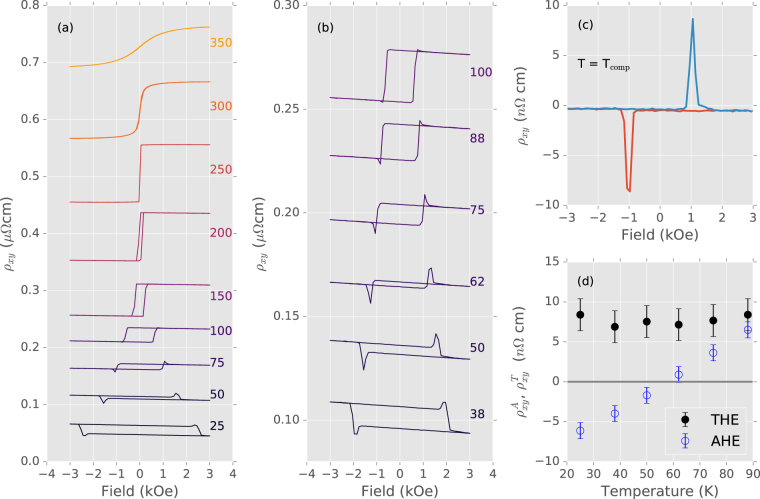
Hall resistivity in MgO/Mn_2_CoAl/Pd trilayers. (**a**) The total Hall resistivity between 25 K and 350 K. The temperature in Kelvin is shown next to each curve, which are plotted with an arbitrary offset for clarity. (**b**) Detailed view around the compensation temperature. (**c**) At exactly the compensation temperature, the contribution of ${\rho }_{xy}^{T}$ stands out. (**d**) Temperature dependance of ${\rho }_{xy}^{T}$ and ${\rho }_{xy}^{A}$ showing an absence of any correlation between these two contributions to *ρ*_*xy*_. ${\rho }_{xy}^{T}$ is defined as the difference between the maximum of the THE peak, and *ρ*_*xy*_ = 0. ${\rho }_{xy}^{A}$ is defined as the difference between *ρ*_*xy*_ at 0 kOe after saturation at 3 kOe and *ρ*_*xy*_ = 0.

We begin by presenting the observation of the topological Hall effect (THE) in Mn_2_CoAl thin films with perpendicular magnetic anisotropy. Hall effect measurements in Fig. 1(a) on a trilayer sample with a Mn_2_CoAl thickness of 1.7 nm measured at temperatures between 375 K and 25 K show all three Hall contributions. The ordinary Hall effect (OHE) is seen in the small linear slope for fields greater than several kOe, where the magnetization is saturated. The slope is negative in all samples. The value of the anomalous Hall effect (AHE), defined as the zero-field extrapolation of the high-field slope, is strongly temperature dependent. In our MgO/Mn_2_CoAl/Pd trilayers it changes from positive to negative at a thickness-dependent compensation temperature (T_comp_), which for the present sample is around 60 K. The topological Hall effect becomes obvious around this compensation temperature [see Fig. 1(b)], appearing as a peak near the coercive field, where the magnetization reverses. Strikingly similar results were recently reported in a SrRuO_3_-SrIrO_3_ bilayer^11^. We take advantage of the lack of AHE at *T*_comp_ to show the pure topological signal in Fig. 1(c).

While the magnitude of the AHE varies quasi-linearly with temperature around the compensation point, Fig. 1(d) shows the topological Hall peak height remains constant at 7 ± 2 nΩcm across the entire temperature range where the peak can be easily distinguished from the other Hall contributions (between 20 K and 90 K for this sample). The independence of ${\rho }_{xy}^{T}$ from ${\rho }_{xy}^{A}$ is interpreted as evidence that the features are of different origins. A change in the magnetization, for instance, would lead to a peak that scaled with the AHE. The temperature independence of the THE further supports its assignment as such: the topological Hall effect scales with ${R}_{0}{B}_{z}^{{\rm{eff}}}$ where *R*_0_ is the OHE coefficient and ${B}_{z}^{{\rm{eff}}}$ is the topological gauge field, neither of which varies strongly with temperature^6^.

**Figure 2:**
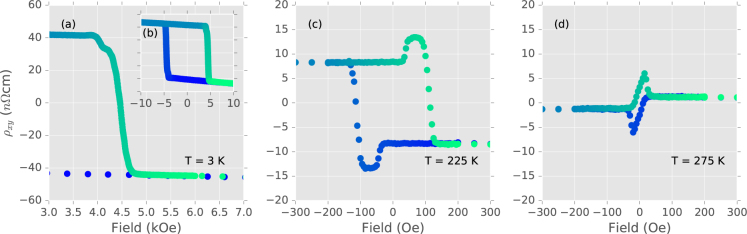
Temperature range of the topological Hall effect. Hall effect measurements on a trilayer with a compensation temperature of 270 K. At 3 K, where the AHE dominates, the THE persists as a shoulder in (**a**,**b**). The THE peaks are clear closer to the compensation temperature in (**c**,**d**), and persists up to almost ambient temperature.

While the topological Hall signal is most obvious around the compensation temperature, a field scan with fine steps of 5 Oe (Fig. 2) shows that it exists over a very wide temperature range, from below 3 K to just under ambient temperature. The trilayer sample shown in Fig. 2 has an AHE compensation temperature around 270 K. Away from the compensation temperature, where the AHE is significant, the topological Hall peak is a shoulder in the measured Hall signal. This is shown in Fig. 2(a,b) at 3 K, where the ${\rho }_{xy}^{A}$ term dominates. The clear ${\rho }_{xy}^{T}$ peaks can be seen closer to the compensation temperature in Fig. 2(c,d). Therefore, the topological magnetic texture responsible for the topological Hall effect exists up to near ambient temperature in this sample. We note that the upper temperature limit here, where the THE vanishes, is the transition to in-plane magnetic anisotropy at around 280 K. This transition can be engineered by varying the thicknesses of the layers in the sample (discussed later). It can thus be assumed that by engineering films where the PMA persists to higher temperatures, the maximum temperature limit for the signature of THE in these films can be increased to room temperature or higher.

## Hall effect minor loops and skyrmion nucleation

Ferromagnetic thin-films with perpendicular magnetic anisotropy can exhibit a magnetic bubble phase between opposite uniformly magnetized states, determined by the competition between the exchange, dipole, and anisotropy energies^22–24^. However, the magnetic bubble phase is topologically trivial, and will not result in an additional contribution to the Hall effect. One must introduce some chirality to the spin texture in order to generate a THE. This approach was successfully demonstrated in Oxide/CoFeB/Ta structures where the spin-orbit coupling of the heavy metallic capping layer provides the required interfacial DMI, leading to the robust formation of a skyrmion bubble phase^10,25^. Given the strong spin-orbit coupling in the heavy Pd layer, we expect the same phenomena are at play in the MgO/Mn_2_CoAl/Pd trilayers studied here, and we therefore interpret the topological Hall signal in Mn_2_CoAl in terms of skyrmion formation. While we cannot rule out the possibility that there is a contribution to the observed THE from other chiral magnetic textures^26^ in the trilayers, the THE reported in similar magnetic multilayer stacks is correlated with the presence of skyrmions^12,27^.

**Figure 3:**
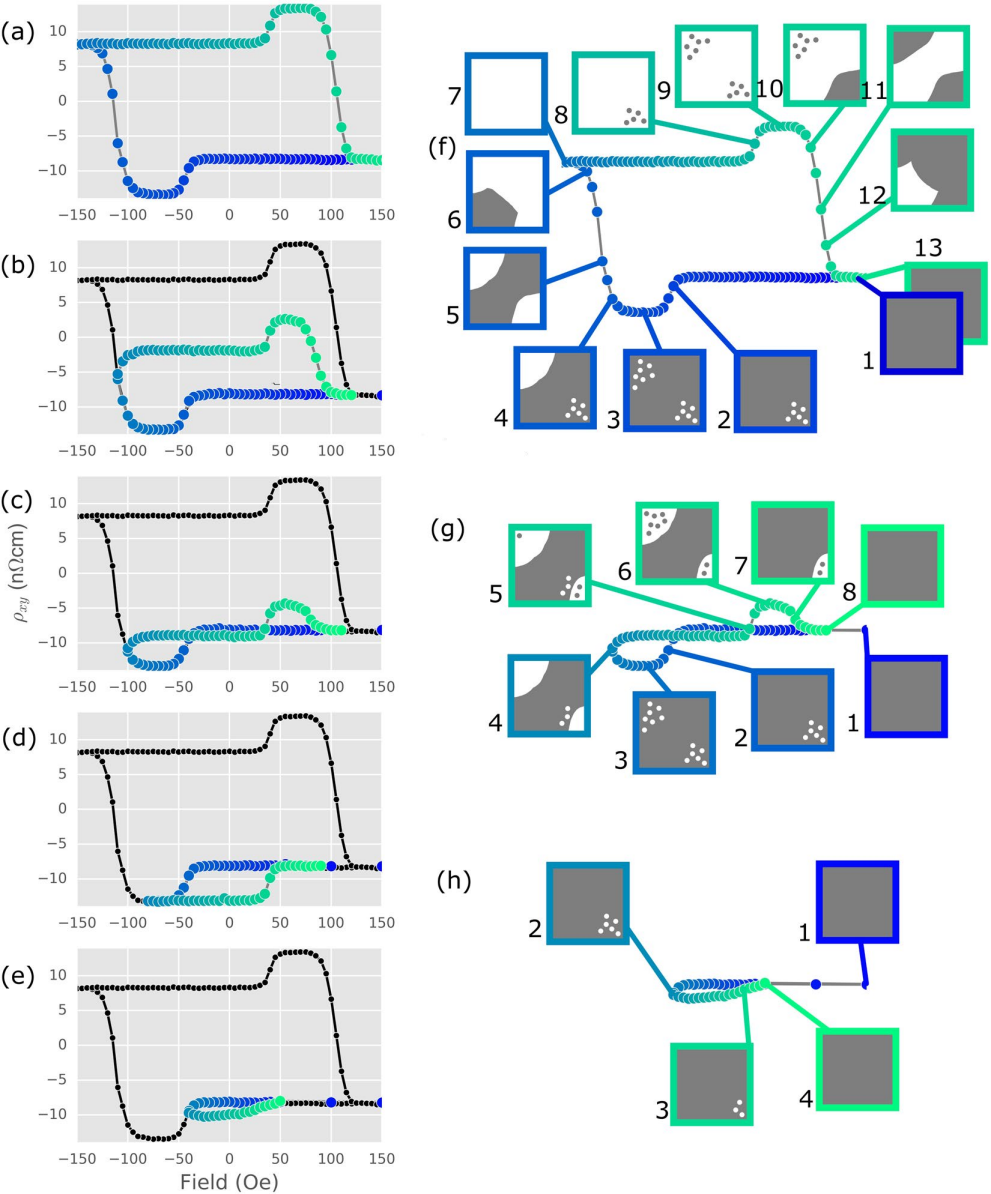
Minor loops are interpreted in terms of skyrmion formation and domain formation. Hall effect measurements at 225 K show both the AHE and THE. The Hall effect is saturated at positive field and the maximum negative field is progressively decreased to measure minor loops in (**a**–**e**). The loops shown in (**a**,**c**,**e**) are interpreted with cartoons in (**f**–**h**) respectively. The solid gray (white) areas represent the ferromagnetic state saturated in positive (negative) applied field, referred to in the text as *FM* ^+^ (*FM* ^−^). Skyrmions are represented by gray (white) points, referred to as *Sk* ^+^ (*Sk* ^−^) in the text. The colour corresponds to the assumed magnetization of the skyrmion core.

In Fig. 3, we demonstrate a novel method to explore the magnetization and skyrmion nucleation processes by studying minor loops of the Hall effect. The measurements were done at 225 K (on a sample with a compensation temperature of 270 K) where both AHE and THE components are obvious. The full field sweep starts at +300 Oe, above the saturation field of the THE and AHE, is swept to the desired negative field and back to positive saturation at +300 Oe. The color represents the sweep direction from blue to green. The Hall resistivity measured in the minor loops shows some significant differences to that measured during the full field sweep, due to the specific magnetic and skyrmionic configurations of the film as the field sweep is stopped and reversed before saturation.

As the magnetization in the ferromagnetic thin film is reversed from full positive saturation (*FM* ^+^) to full negative saturation (*FM* ^−^), it passes through two intermediary phases. First, a skyrmion phase, where skyrmions with negative magnetization at the center (*Sk* ^−^) are nucleated in the *FM* ^+^ background. This is followed by by a stripe or labyrinth domain phase (*D*), before the uniform ferromagnetic phase with reversed magentization (*FM* ^−^)^23,28,29^. We can denote this process as *FM* ^+^ → *Sk* ^−^ → *D* → *FM* ^−^.

The full loop in Fig. 3(a) can be described by this model, which we do with the aid of cartoons shown in Fig. 3(f). In the cartoons, grey (white) represents magnetization along the positive (negative) applied field direction. The solid regions represent domains, while the small circles represent skyrmions (with the color corresponding to the core magnetization). The film starts in a uniformly magnetized *FM* ^+^ condition at +150 Oe (Fig. 3(f), position 1), corresponding to a saturated magnetization and corresponding Hall resistivity of −9 nΩcm. The field is swept down to approximately −30 Oe, where an additional negative contribution to the Hall resistivity appears due to the presence of a THE, indicating that *Sk* ^−^ skyrmions start to form (position 2). A plateau of Hall resistivity is then reached, where the skyrmion phase has reached its maximum extent (position 3). Sweeping the field further into negative values, we see the Hall resistivity start to change as the magnetization of the film is reversed (positions 4–6), simultaneously annihilating the *Sk* ^−^ skyrmions. Saturation is reached at −150 Oe, where the film is uniformly magnetized in the direction of the (negative) field (position 7). Upon sweeping the field back from −150 Oe to +150 Oe, the same process occurs, except this time the onset of the THE (position 8) is shown by an additional positive contribution to the Hall resistivity, which appears at positive fields. This corresponds to the appearance of *Sk* ^+^ skyrmions in the now reversed uniform ferromagnetic phase *FM* ^−^. The positive THE then saturates (position 9), and is then eliminated as the film magnetization reverses and saturates at +150 Oe (positions 10–12).

The minor loops in Fig. 3(b–e) are also well described in this simple picture. In Fig. 3(b), the downward field sweep is stopped after the peak of the negative THE and in the *D* domain phase where only partial reversal has occured (position 5). Sweeping the field from this position in the positive sense, the *D* phase is present until the THE peak appears, at the same positive field as it does during the full field sweep of Fig. 3(a) (position 8). The appearance of the positive THE peak during the field sweep up at +30 Oe shows that the *Sk* ^+^ skyrmions can nucleate when the film is in the *D* phase reached at position 5.

In Fig. 3(d), the downward field sweep is stopped at the peak of the negative THE. Sweeping the field upward from this point shows that the full magnitude of the THE persists through zero field. At higher positive fields the THE vanishes once again. In Fig. 3(e), which is explained with cartoons in Fig. 3(h), the downward sweep is stopped just after the onset of the THE peak (position 2 of Fig. 3(h)), where the negative THE component has appeared but has not reached its maximum, and the field is not sufficiently negative to have reached the *D* phase. Sweeping the field in the positive direction from this position again shows that *Sk* ^−^ skyrmions persist through zero field, as shown by the Hall resistivity being larger (more negative) than its value with the film saturated at positive field. Skyrmions have been observed to shrink in a magnetic field applied opposite to the skyrmion core polarity^12,30^, which is likely the mechanism of skyrmion annihilation in this minor loop.

The intermediate minor loop in Fig. 3(c), explained with cartoons in Fig. 3(g), implies that both skyrmion polarities (*Sk* ^+^ and *Sk* ^−^) can coexist in a carefully conditioned sample. In the minor loop of Fig. 3(c), the sweep to negative field is now stopped just at the start of the rise in Hall resistivity (position 4 of Fig. 3(g)) showing that the film magnetization has reached the *D* phase, and some *Sk* ^−^ skyrmions are being annihilated. Upon sweeping the field in the positive direction, the Hall resistivity is more negative than the saturation value reached at large positive fields. This is a clear sign that, during the up sweep through zero field, there is still a contribution from the negative THE, implying that some *Sk* ^−^ skyrmions still exist in the film. Between positions 4 and 5 there is a positive contribution to the Hall resistivity, taking its value back to that of the *FM* ^+^ state. This could come from the annihilation of the remaining *Sk* ^−^, or the nucleation of *Sk* ^+^ in the *FM* ^−^ regions of the film. The former possibility would lead to a skyrmion-free mixed domain state at position 5. We can rule out this possibility, as such a mixed domain state would have a Hall resistance smaller (less negative) than that of the *FM* ^+^ state in positions 1 and 8. At position 5, we therefore infer the coexistence of *Sk* ^+^ and *Sk* ^−^ in different regions of the film. Between positions 5 and 6, the remaining *Sk* ^−^ are annihilated, and *Sk* ^+^ are nucleated. Further sweeping the field in the positive direction results in the positive THE signal disappearing (position 7) and the Hall resistivity reaches its positive field saturation value at position 8, as in the full field sweep. A key point is that *Sk* ^+^ can only nucleate in regions of the film where the magnetization had reversed to *FM* ^−^ (ie. white regions). This provides a natural explanation for the peak height being the same in both sweep directions: *Sk* ^−^ are annihilated by the reversal of the surrounding magnetization (position 4), and these regions then support the exact same number of *Sk* ^+^ at positive field (position 6).

Our minor loop data clearly imply that we have a non-uniform film, where some regions have the Goldilocks combination of magnetic anisotropy and DMI to support skyrmions, while other regions do not. These other regions will undergo a magnetization reversal process *FM* ^+^ → *D* → *FM* ^−^, seen in the Hall measurement as a change in the AHE only. In Fig. 3(b), the THE peak height is the same in both sweep directions, although the magnetization has not fully reversed. This implies that all the regions that do support skyrmions have reversed, and can therefore support the same number of opposite polarity skyrmions on the up-sweep. Meanwhile, the remainder of the film is in a mixed domain state, leading to a non-saturated AHE. The apparent reduced width of the THE peak on the up-sweep is interpreted as a reduction in the field required to saturate the magnetization from this mixed domain state.

The minor loops show that once the skyrmions appear in the films, they persist until the field where the magnetization of the films begins to reverse, where domain reversal eliminates them and the film magnetization becomes uniform. Skyrmions can thus be nucleated in the trilayer and are robust against the field being reduced to 0 Oe, and they even survive to small reverse field values of order 10 s of Oe. These results also demonstrate that skyrmions of opposite sign may simultaneously exist at a given temperature and applied field, in different regions of the film. Our picture of the coexistence of opposite polarity skyrmions could have important implications for current-driven motion of skyrmions in device for memory storage. To confirm this picture and establish the details of the phase that allow the topological Hall effect to emerge, it will be useful to carry out follow-up investigations of the magnetic structure at the micro- and nano-scale, such as magnetic force microscopy or magnetic transmission x-ray microscopy^12,13^.

## Tuning AHE and PMA

We now return to discuss the material and multilayer properties that enable the existence and detection of the topological Hall effect. The THE is a small signal compared to the anomalous Hall effect (${\rho }_{xy}^{A}$) and can often be hard to separate. Previous reports of the skyrmion related topological Hall effect have relied on the subtraction of a term proportional to the magnetization from the Hall resistance to elucidate the topological contribution^11,12,19,31^. Here, we use a different approach, physically tuning the AHE to exactly zero by leveraging it’s strong Mn_2_CoAl thickness and temperature dependence. We show in the Supplementary Information that the two approaches yield similar results. The magnitude of the AHE, while proportional to the spin polarization, is determined by the sum of three spin-orbit derived mechanisms: the skew-scattering, side-jump, and intrinsic terms^32^. These terms determine the scaling with the longitudinal resistivity (and hence also temperature), typically ∝${\rho }_{xx}^{\beta }$ with 1 < *β* < 2^33^. The film thickness and interfaces also influence the constituent terms of the AHE^11,34^.

**Figure 4:**
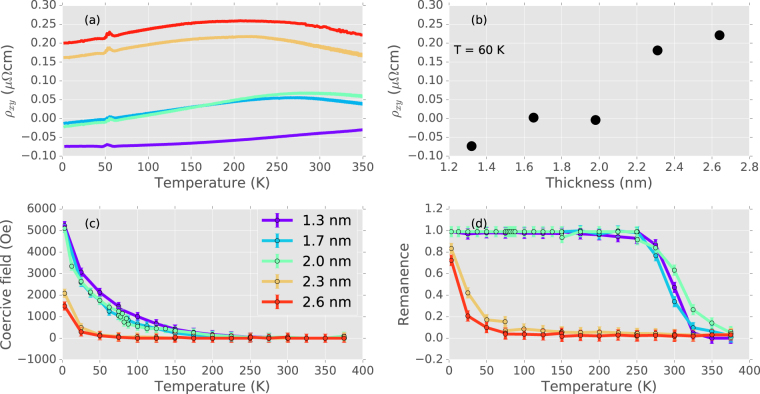
Tuning the AHE and PMA in MgO/Mn_2_CoAl/Pd trilayers. (**a**) Temperature dependence of ${\rho }_{xy}^{A}$ for samples of different Mn_2_CoAl thickness (the MgO and Pd layers are fixed at 2 and 2.5 nm respectively). The bump around 50 K in all samples is an artifact due to a change in the slope of the ordinary Hall effect around this temperature. (**b**) At 60 K, ${\rho }_{xy}^{A}$ increases with increasing Mn_2_CoAl film thickness. The coercive field and remanent magnetization measured with the applied field perpendicular to the sample plane in (**c**,**d**) demonstrate PMA for films below 2 nm thick up to ambient temperature.

We use the film thickness, interface layers and temperature scaling to suppress the AHE in Mn_2_CoAl. The AHE determined from Hall effect measurements for a series of trilayer samples with varying Mn_2_CoAl thickness is shown in Fig. 4(a). The AHE scales with temperature with a thickness dependent offset, and the compensation temperature of the AHE can be tuned into the accessible temperature range by changing the thickness of the Mn_2_CoAl film. The AHE reaches zero for two of the samples in this series, with nominal Mn_2_CoAl thicknesses of 1.7 and 2.0 nm. The strong thickness dependence of the AHE at 60 K is shown in Fig. 4(b), where ${\rho }_{xy}^{A}$ increases from negative to positive values with increasing Mn_2_CoAl thickness. This hints at a possible competition between contributions to ${\rho }_{xy}^{A}$ from the bulk and interface.

The existence, size, and density of skyrmions in a thin film or interfacial system are controlled by the interplay of the PMA, and the DMI^10^. The DMI is set largely by the choice of capping layer, but the PMA can be tuned. In magnetic thin-films and multilayers, the magnetic anisotropy is determined by the competition between the bulk, which tends to favor in-plane magnetization, and the interfaces, which favor PMA. The magnetic anisotropy can be tuned by the film thickness and choice of interfaces. This has enabled the engineering of PMA in a variety of magnetic materials, including Heusler alloys, by sandwiching the magnetic layer between MgO and a heavy metal capping layer^34–37^. We follow this strategy, employing Pd as the capping layer material. We observe PMA only in the thinner Mn_2_CoAl films, shown by the perpendicular field magnetic remanence and coercive field in Fig. 4(c,d), where the interfacial PMA dominates. The Pd layer, with its significant spin-orbit coupling, contributes to both the interfacial PMA and the interfacial DMI, and is critical to the formation of the skyrmion phase.

In summary, the topological Hall signal is observed over a broad temperature range in Mn_2_CoAl thin films with PMA, from below 3 K to near ambient temperature. We infer from these results the existence of skyrmions in these films over the same extended temperature range. We present Hall effect minor loops, interpreted with a simple model to explain how the ferromagnetic phase is inverting by passing through a skyrmionic bubble phase. This novel approach provides a handle for understanding the magnetization and skyrmion nucleation processes by purely electronic means.

## Methods

Thin films were grown by DC magnetron sputtering in a Kurt J Lesker CMS-18 UHV system with a base pressure of 2 × 10^−8^ Torr. Multilayer stacks were prepared on 10 × 10 mm Si (+native oxide) substrates in the sequence MgO(2)/Mn_2_CoAl(t)/Pd(2.5), where the number in parentheses is the nominal layer thickness in nanometers. Samples were grown at ambient temperature and post-growth annealed *in*-*situ* for 1 hour at 300 °C. Hall effect measurements were made on samples patterned into Hall bars (l = 1600, w = 150 *μ*m) using photolithography, and dry etching or lift-off, and on unpatterned samples in a Van der Pauw geometry. The composition of the Heusler target was verified to be Mn_2_CoAl by energy dispersive x-ray analysis in a SEM. Structural characterization of the thin films was not possible, however, XRD on thicker films indicates a high level of disorder.

For transport measurements, we used a Quantum Design Physical Property Measurement System (PPMS) with the resistivity option. Samples were mounted using a sample holder from Wimbush Science and Technology, with spring-loaded contacts. Typical bias current used on the device was 100 *μ*A. All samples were measured in a similar way, with complete magnetic hysteresis loops measured between 3 T and −1 T, at angles of −90° and 90° relative to the sample plane at a set of temperatures (375 K, 250 K, 150 K, 75 K and 3 K). The temperature ramp rate was kept at a constant 3 K/min. During cool down (warm up), the sample was kept at 3 T (7 T) and measured to obtain a temperature dependent measurement of the AHE between 375 K and 3 K. Finally each sample was measured in detailed magnetic hysteresis loops between +3000 and −3000 Oe every 25 K between 375 K and 75 K, and between +4000 and −4000 Oe at 25 K and 50 K. Additional measurements were made at temperatures near interesting features such as the compensation of the AHE.

The dataset generated during and/or analysed during the current study are available from the corresponding author on reasonable request.

## Electronic supplementary material

Supplementary Information
