# Supplementary material for: Nucleation and annihilation of skyrmions in Mn2CoAl observed through the topological Hall effect
Source: Sci Rep. 2017 Oct 19;7:13620. doi: 10.1038/s41598-017-13211-8 (PMC5648899; doi:10.1038/s41598-017-13211-8)
Supplement: Supplementary file 1 — Supplementary Information [file 41598_2017_13211_MOESM1_ESM.pdf]

Supplementary Material: Nucleation and annihilation of skyrmions in Mn_2CoAl observed through the topological Hall effect

B. M. Ludbrook[1], G. Dubuis[2], A.-H. Puichaud[2], B. J. Ruck[1] & S. Granville[2]

[1] *The MacDiarmid Institute for Advanced Materials and Nanotechnology, School of Chemical and Physical Sciences, Victoria University of Wellington, P.O. Box 600, Wellington 6140, New Zealand*

[2] *The MacDiarmid Institute for Advanced Materials and Nanotechnology, Robinson Research Institute, Victoria University of Wellington, P.O. Box 33436, Lower Hutt 5046, New Zealand*

Methods

Thin films were grown by DC magnetron sputtering in a Kurt J Lesker CMS-18 UHV system with a base pressure of 2×10^{-8} Torr. Multilayer stacks were prepared on 10×10 mm Si (+ native oxide) substrates in the sequence MgO(2)/Mn_2CoAl(t)/Pd(2.5), where the number in parentheses is the nominal layer thickness in nanometers. Samples were grown at ambient temperature and post-growth annealed in-situ for 1 hour at 300 °C. Samples were patterned into Hall bars (l = 1600, w = 150 μm) using photolithography, and dry etching or lift-off. The composition of the Heusler target was verified to be Mn_2CoAl by energy dispersive x-ray analysis in a SEM. Structural characterization of the thin films was not possible, however, XRD on thicker films indicates a high level of disorder.

For transport measurements, we used a Quantum Design Physical Property Measurement System (PPMS) with the resistivity option. Samples were mounted using a sample holder from Wimbush Science and Technology, with spring-loaded contacts. Typical bias current used on the device was 100 μA. All samples were measured in a similar way, with complete magnetic hysteresis loops measured between 3 T and -1 T, at angles of -90° and 90° relative to the sample plane at a set of temperatures (375 K, 250 K, 150 K, 75 K and 3 K). The temperature ramp rate was kept at a constant 3 K/min. During cool down (warm up), the sample was kept at 3 T (7 T) and measured to obtain a temperature dependent measurement of the AHE between 375 K and 3 K. Finally each sample was measured in detailed magnetic hysteresis loops between $+3000$ and -3000 Oe every 25 K between 375 K and 75 K, and between $+4000$ and -4000 Oe at 25 K and 50 K. Additional measurements were made at temperatures near interesting features such as the compensation of the AHE.

Transmission Electron Microscopy

The transmission electron microscopy (TEM) sample was prepared using conventional cross-section sample preparation. The sample was coated with an epoxy resin and a silicon chip was attached to the thin film side to protect it during grinding and milling stages. The stack was cured for 30 minutes at 120 °C, then mounted onto a tripod polisher and ground to approximately 30 m thick using diamond grinding films. The sample was then mounted onto a TEM grid and thinned to electron transparency using a Gatan Precision Ion Polishing System operated at 4-2 keV. The samples were investigated in a JEOL2100F TEM which is equipped with a field emission gun and

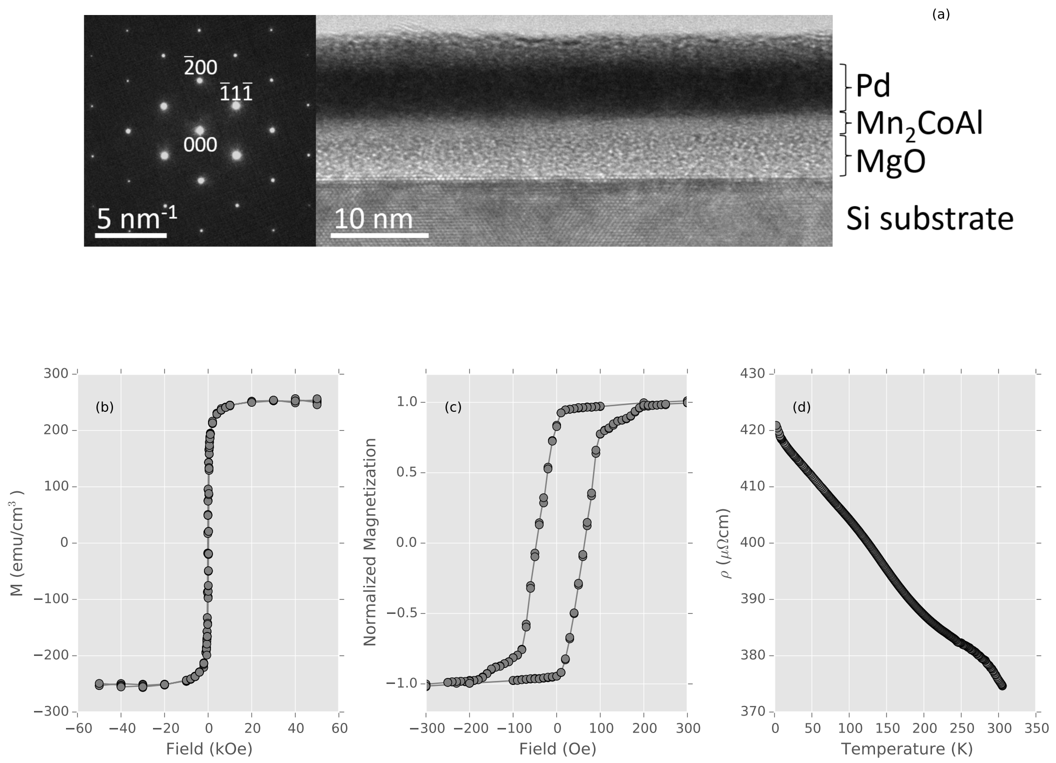

Figure 1: The structure of the trilayer stack is confirmed in a TEM image in (a). The magnetization of a thick Mn_2CoAl layer (33nm) at 10 K is shown in (b). The magnetization measured with the field applied in-plane along the easy axis saturates at the the expected bulk value. Figure (c) shows the magnetization at 200 K for a trilayer sample measured with field out-of-plane: this sample is the same as the one of Figures 2 and 3 in the main article. Over the field range where skyrmions occur, the magnetization loop shows a characteristic shearing. Figure (d) shows the resistivity of a thick Mn_2CoAl layer. The resistivity increases with decreasing temperature, which has been attributed to the spin-gapless semiconducting band structure of the material.

a JEOL SDD (silicon drift detector) EDS (energy dispersive spectroscopy) detector. The TEM was operated at 200 keV. The TEM is equipped with a high-resolution charge-coupled device (CCD) camera for high-resolution image acquisition and lower resolution higher dynamic range CCD for diffraction acquisition. Images were collected in Digital Micrograph which was also used for image processing.

While we cannot rule out some interdiffusion at the interfaces, no evidence for secondary phases was detected in the TEM image of Fig. 1(a) or from X-ray diffraction (not shown).

Magnetization and resistivity properties of Mn_2CoAl film and trilayers

The longitudinal resistivity of a 33 nm thick Mn_2CoAl film with MgO capping layer increases with decreasing temperature as shown in Fig. 1(d). This behavior is consistent with previous reports in bulk and sputtered thin-film samples, where it has been interpreted in terms of the spin gapless semiconducting (SGS) behavior .[1,2]

This same sample measured with the field in-plane (Fig. 1(b)) has a saturation magnetization of about 250 emu/cm^3. This corresponds to $\sim$1.3 μ_B/f.u., which is lower than the expected $\sim$2 μ_B per formula unit in bulk Mn_2CoAl [1], but reasonable compared to the $\sim$1 μ_B per formula unit of thin films reported previously in the literature. [3,4]

The magnetization of a trilayer sample measured at 200 K with field out-of-plane is shown in Fig. 1(c), normalised to its saturation magnetization. This sample is the same as that of Figures

2 and 3 in the main text, which show a clear topological Hall effect. The magnetization loop of Fig. 1(c) shows the characteristic shearing that accompanies skyrmion formation. [5,6] Moreover, the shearing occurs from ∼80-200 Oe, covering the same field range where the topological Hall effect appears in this sample.

In summary, although these films have not been structurally optimized, the electronic and magnetic properties of thick films are in good agreement with previous reports, and the trilayer magnetization loop confirms the presence of skyrmions.

PMA in thin films

A series of trilayers (MgO/Mn_2CoAl/Pd) was grown with incremental increases in the Mn_2CoAl thickness. The Hall effect measurements for each sample are shown in Fig. 2. The color represents the temperature, and the curves have been offset for clarity. In the Hall effect measurement geometry, the magnetic field is applied perpendicular to the film plane, and the AHE is proportional to M_z, the perpendicular magnetization. The high remanent magnetization of the square Hall loops at lower temperature and in thinner films is evidence of a perpendicular magnetic easy axis. At temperatures above around 275 K and in the two thickest samples, M_Z vanishes as the applied field is reduced to zero, as the perpendicular axis is no longer the easy axis. This behaviour is indicative of interfacial-PMA, where the interfacial magnetic anisotropy favours the perpendicular direction, and dominates in thin films, while the bulk magnetic anisotropy favours in-plane, and dominates for thicker films.

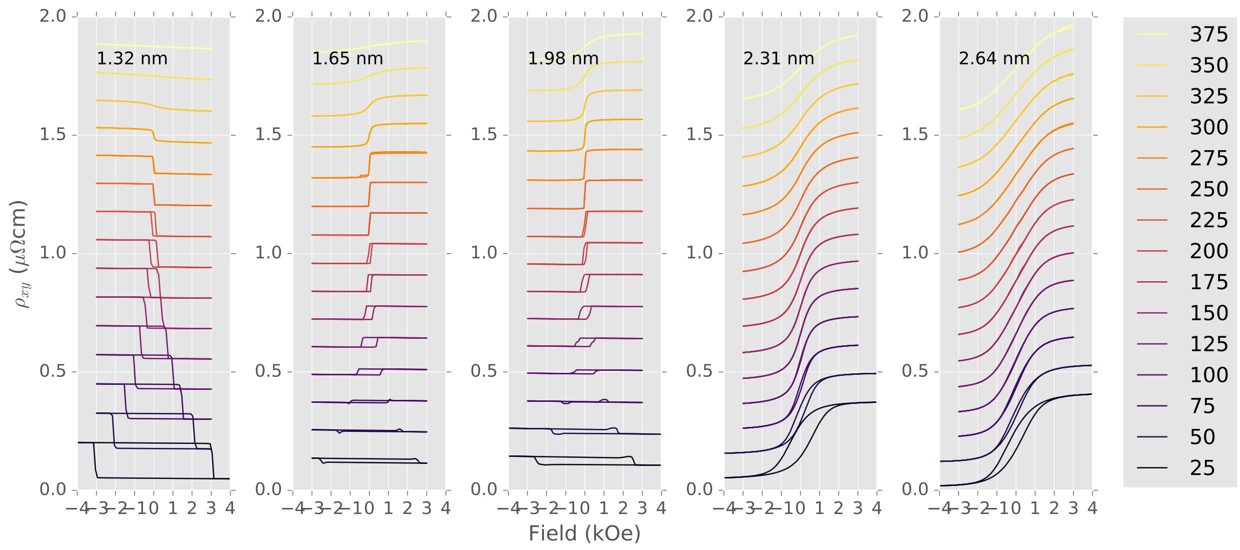

Figure 2: Magnetic properties of five films with different thicknesses. The abscissa shows the magnetic field while the ordinate shows the Hall resistance with an arbitrary offset for better legibility. All samples were measured at 250 K, 150 K, 75 K and 3 K. The hysteresis loops clearly show the AHE in all samples. AHE is positive (resp. negative) for the thin (resp. thick) samples at 250K. It is remarkable that the sign of the AHE changes for samples 2 and 3 (1.7 nm and 2.0 nm) as they are cooled down. The THE can be seen is sample 2 and 3 in form of a peak near the edge of the hysteresis loop. This is most visible at the temperature at which the AHE is canceled out.

Cancellation of AHE

We present data for three samples that show a sign change in the AHE with temperature in Figs. 3(a)-(c). The 1.5 nm thick sample in Fig. 3(a) is not patterned into a Hall bar like the others, and the Hall effect measurements were done in a Van der Pauw geometry. This helps to establish that there are no artifacts arising from the geometry of the Hall bar or from the sample processing.

At the compensation temperature, the Hall signal consists of just the topological Hall effect and the linear OHE. The linear OHE is fitted at positive field and subtracted from the data in Figs. 3(d)-(e) allowing the unambiguous observation of the pure topological Hall effect for different film thicknesses.

In previous reports, the THE has been extracted from the total Hall signal by subtracting the AHE, which is expected to be proportional to the magnetization. [6–9] Fig. 4 shows the result of the same subtraction process to reveal the THE of the trilayer of main text Figs. 2 and 3. While noting that Fig. 4 is from a different THE-containing sample, the shape of the THE extracted in this manner is similar to the THE shape in Figs. 3(d)-(f), confirming that our approach of tuning the AHE to zero at the compensation temperature is able to reveal the THE signal without relying on separate magnetization measurements.

1. Ouardi, S., Fecher, G. H., Felser, C. & Kübler, J. Realization of spin gapless semiconductors: The Heusler compound Mn 2CoAl. *Phys. Rev. Lett.* **110**, 100401 (2013). URL http://link.aps.org/doi/10.1103/PhysRevLett.110.100401.

2. Xu, G. Z. *et al.* Magneto-transport properties of oriented Mn2CoAl films sputtered on thermally oxidized Si substrates. *Appl. Phys. Lett.* **104**, 242408 (2014). URL http://aip.scitation.org/doi/10.1063/1.4884203.

3. Jamer, M. E., Assaf, B. A., Devakul, T. & Heiman, D. Magnetic and transport properties of Mn_2CoAl oriented films. *Appl. Phys. Lett.* **103**, 142403 (2013). URL http://link.aps.org/doi/10.1103/PhysRevLett.98.207205.

4. Sun, N. Y. *et al.* Perpendicular magnetic anisotropy in Mn 2 CoAl thin film. *AIP Adv.* **6**, 015006 (2016). URL http://aip.scitation.org/doi/10.1063/1.4939934.

5. Woo, S. *et al.* Observation of room-temperature magnetic skyrmions and their current-driven dynamics in ultrathin metallic ferromagnets. *Nature Materials* **15**, 501–506 (2016). URL http://www.nature.com/doifinder/10.1038/nmat4593.

6. Soumyanarayanan, A. *et al.* Tunable Room Temperature Magnetic Skyrmions in Ir/Fe/Co/Pt Multilayers. *Nat. Mater.* **advance online publication** (2017). URL http://dx.doi.org/10.1038/nmat4934.

7. Liang, D., Degrave, J. P., Stolt, M. J., Tokura, Y. & Jin, S. MnSi nanowires revealed by topological Hall effect. *Nature Communications* **6**, 1–8 (2015). URL http://www.nature.com/doifinder/10.1038/ncomms9217 http://dx.doi.org/10.1038/ncomms9217.

8. Rana, K. G. *et al.* Observation of topological Hall effect in Mn2RhSn films. *New J. Phys.* **18**, 085007 (2016). URL http://iopscience.iop.org/article/10.1088/1367-2630/18/8/085007/meta.

9. Matsuno, J. *et al.* Interface-driven topological Hall effect in SrRuO3-SrIrO3 bilayer. *Science Advances* **2**, e1600304 (2016). URL http://advances.sciencemag.org/content/2/7/e1600304.

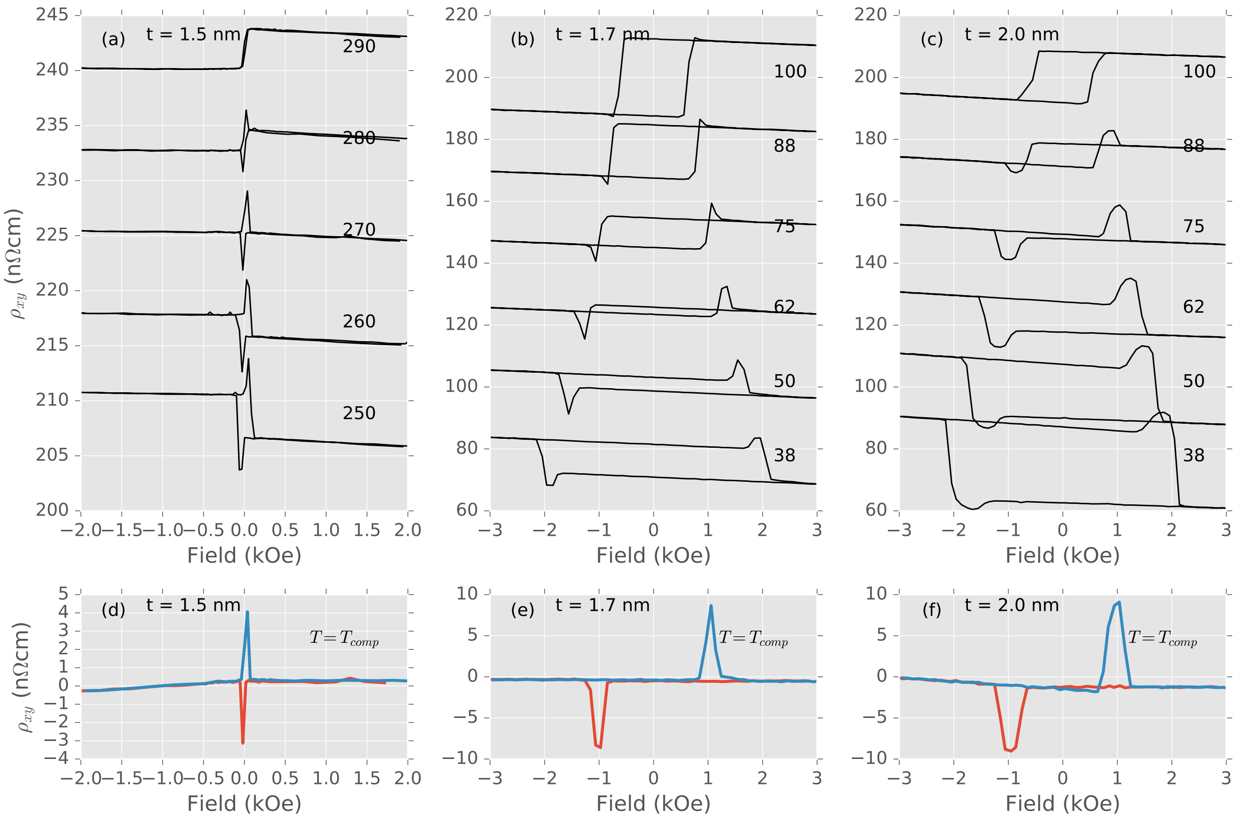

Figure 3: Data near the AHE compensation temperature for three thicknesses of Mn_2CoAl. The 1.5 nm thick sample in (a) is un-patterned, and the Hall effect was measured in a Van der Pauw geometry. The samples in (b) and (c) were patterned into Hall bars, and form part of the series of samples shown in Fig. 2. The pure topological signal at the compensation temperature is shown in (d)-(e) where the red (blue) curve is for decreasing (increasing) field.

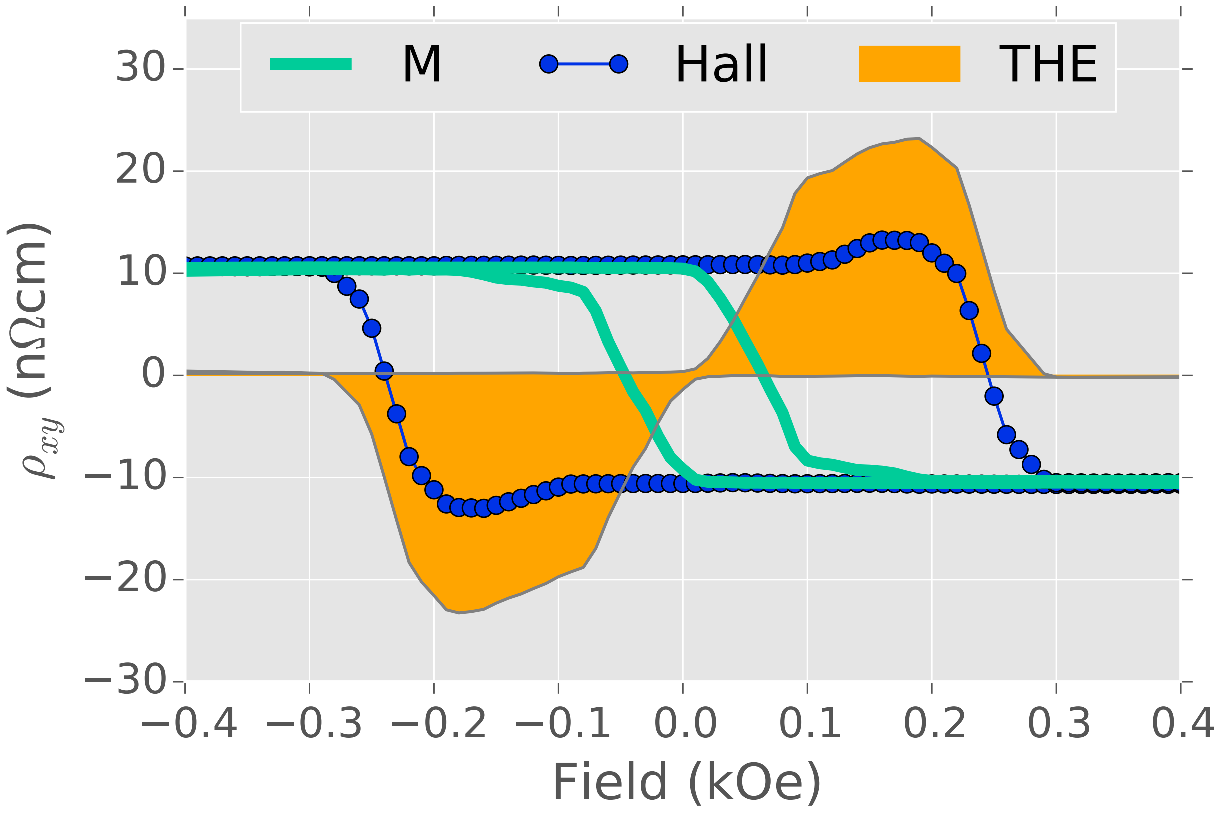

Figure 4: Contribution from the THE separated from the measured (total) Hall resistivity by subtraction of a component proportional to the magnetization M (i.e. the AHE). M is shown reversed compared to Fig. 1(c) to account for the opposite sign of the AHE. Measurements were done at 200 K.
